# Artesunate Misuse and *Plasmodium falciparum* Malaria in Traveler Returning from Africa

**DOI:** 10.3201/eid1610.100427

**Published:** 2010-10

**Authors:** Dea Shahinas, Rachel Lau, Krishna Khairnar, David Hancock, Dylan R. Pillai

**Affiliations:** Author affiliations: University of Toronto, Toronto, Ontario, Canada (D. Shahinas, K. Khairnar, D.R. Pillai);; Ontario Agency for Health Protection and Promotion, Toronto (R. Lau, D.R. Pillai);; Ajax-Pickering Hospital, Ajax, Ontario, Canada (D. Hancock)

**Keywords:** Malaria, Plasmodium falciparum, parasites, artemisinin, travel, Africa, dispatch

## Abstract

*Plasmodium falciparum* malaria developed in an African-born traveler who returned to Canada after visiting Nigeria. While there, she took artesunate prophylactically. Isolates had an elevated 50% inhibitory concentration to artemisinin, artesunate, and artemether, compared with that of other African isolates. Inappropriate use of artemisinin derivatives can reduce *P. falciparum* susceptibility.

Artemisinin derivatives were recently approved by the Food and Drug Administration for the treatment of *Plasmodium falciparum* malaria in North America and are available through the US Centers for Disease Control and Prevention and through Health Canada (*1**–**3*). Artemisinin-based combination therapy (ACT) remains the most effective therapy for *P. falciparum* malaria throughout the world, with the possible exception of the Thailand–Cambodia border (*4*). Because of the large numbers in the Toronto area of returning travelers and recent immigrants who have returned to countries of origin and visited friends and relatives, the Public Health Laboratory (Toronto) identifies ≈200 positive malaria smears annually; most *P. falciparum* isolates have come from sub-Saharan Africa. Evidence has indicated that such travelers tend not to seek medical advice before travel and are therefore at high risk of acquiring malaria (*5*).

## The Patient

A 38-year-old Nigerian-born woman, who lived in the Toronto area (and has a good ability to recount her experiences), returned to Lagos, Nigeria, for a visit in January 2009. She did not seek pretravel advice. On arrival in Lagos, the woman purchased artesunate locally and began taking two 50-mg tablets weekly for the 4 weeks of her visit. Immediately on her return to Toronto, the patient experienced myalgia, nausea with vomiting, and chills, ≈7 days after she had taken her last dose of oral artesunate. She sought treatment at the emergency department of a community hospital. Physical examination showed that her temperature was 39.1°C and that she was dehydrated. Laboratory tests showed the following: leukocyte count 3,700 cells/μL, thromocyte count 72 × 10^3^ cells/μL, hemoglobin level 12.7 g/dL. Her chest radiograph showed that her lungs were clear. An examination of peripheral blood by thick and thin films showed a 0.7% parasitemia with *P. falciparum*. Her condition was treated with 1,250 mg of oral mefloquine as a single dose. She was treated as an outpatient, and she reported that symptoms promptly resolved over the next 48 hours without side effects.

A blood specimen was placed into culture in the Public Health Laboratory (Toronto), and the *P. falciparum* isolate was tested for drug susceptibility (*6*). The 50% inhibitory concentration (IC_50_) was the following for certain antimicrobial agents (tested in triplicate): chloroquine 170.5 ± 7.8 nmol/L, mefloquine 16.6 ± 0.7 nmol/L, artemisinin 20.1 ± 0.6 nmol/L, artesunate 6.2 ± 1.4 nmol/L, dihydroartemisinin 1.8 ± 0.9 nmol/L, and artemether 21.4 ± 5.3 nmol/L. For this *P. falciparum* isolate, IC_50_ was significantly higher for artemisinin, artesunate, and artemether than for other representative *P. falciparum* isolates imported from Africa (Figure). Because of the short half-life of artesunate, the weekly doses of the oral drug may have led to development of a resistant strain when the patient was in Nigeria. Artesunate-containing drugs therefore should not be used for prophylaxis or single drug therapy. The purchased artesunate may also have been counterfeit and may have contained lower levels of active drug. Although these data suggest that this isolate has reduced susceptibility to artemisinin derivatives, the correlation between in vitro susceptibility and treatment outcomes does not appear to be consistent (*4*).

**Figure:**
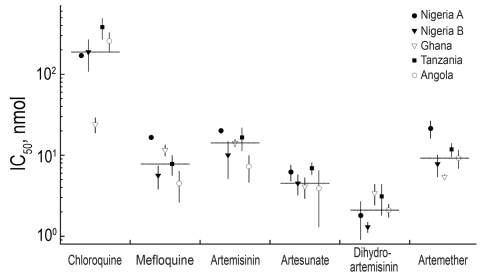
In vitro drug susceptibility of representative patient isolates from returning travelers who visited friends and relatives in Africa. The mean 50% inhibitory concentrations (IC_50_) of chloroquine, mefloquine, artemisinin, artesunate, dihydroartemisinin, and artemether are plotted in nmol/L for each isolate, performed in triplicate (error bars indicate SD; n = 3). Nigeria A denotes the patient described in this report. The black horizontal line represents the median value.

Previous studies have reported that resistance to artemisinin is mediated by an increase in gene copy number, mutations within the efflux pump of the *P. falciparum* multidrug resistance 1 (*pfmdr1*) gene, or mutations in the calcium transporter *pfATPase6* (*7**,**8*). When we examined each gene, using a combination of real-time PCR and DNA sequencing, we found that *pfmdr1* copy number was elevated in this isolate relative to that of the susceptible control strain 3D7. We also observed nonsynonymous mutations in both *pfmdr1* (Y184F) and *pfATPase6* (A623E, S769N), whereas other implicated residues remained in the wild-type form (*9*) (Table). Similar molecular analysis of other representative imported African clinical isolates demonstrated variable mutations for *pfmdr1* and *pfATPase6* and copy number in relation to IC_50_ values for key drugs (Table). A trend, albeit weak, was observed in which increased *pfmdr1* copy number was correlated with an elevated IC_50_ to mefloquine (*r* = 0.52) and artemisinin (*r* = 0.42). The presence of an asparagine (N) at position 86 of Pfmdr1, when coupled to an elevated *pfmdr1* copy number, appeared to correlate well with reduced susceptibility to artemisinin (Table). Chavchich et al. recently demonstrated that increased *pfmdr1* copy number occurred in a laboratory strain placed under drug selection pressure with artemisinin derivatives (*11*). However, Imwong et al. have indicated that genetic polymorphisms and copy number in *pfmdr1* do not predict treatment outcome with ACT (*10*).

**Table:** Results of sequencing single-nucleotide polymorphisms of *Plasmodium falciparum* isolate*

Strain	Pfmdr1						PfATPase		*pfmdr1* copy no.	CQ IC_50_, nmol/L	MQ IC_50_, nmol/L	ART IC_50_, nmol/L
	86	184	1034	1042	1246		623	769				
3D7	N	Y	S	N	D		A	S	1.00	6.1	2.1	6.1
W2	Y	Y	S	N	D		A	S	0.97	252	3.2	7.3
Cameroon	Y	F	S	N	D		E	N	1.85	163	7.7	8.07
Congo	Y	F	S	N	D		E	N	1.51	355	10.7	10.9
Kenya	Y	F	S	N	D		E	N	1.75	282	11.7	10.1
Liberia	N	F	S	N	D		E	N	1.65	109	16.2	16.5
Nigeria C	Y	F	S	N	D		E	N	1.06	222	8.7	8.1
Nigeria A	N	F	S	N	D		E	N	1.52	171	16.6	20.1
Nigeria B	Y	F	S	N	D		E	N	1.09	188	5.6	10.0
Ghana	N	F	S	N	D		E	N	0.96	24.0	11.6	14.2
Tanzania	Y	F	S	N	Y		E	N	1.88	381	7.8	16.6
Angola	Y	F	S	N	D		E	N	0.81	258	4.5	7.3

* At Pfmdr1 and PfATPase6 residues previously implicated in artemisinin resistance and gene copy number of *pfmdr1* by quantitative real-time PCR in relation to mean IC_50_ (n = 3) data for key drugs (*7**,**8**,**10*). *pfmdr1*, *P. falciparum* multidrug resistance 1; CQ, chloroquine; MQ, mefloquine; ART, artemisinin; IC_50_., 50% minimum inhibitory concentration; N, asparagine; Y, tyrosine; S, serine; D, aspartic acid; A, alanine; E, glutamic acid; 3D7, chloroquine-sensitive laboratory strain; W2, chloroquine-resistant laboratory strain; Nigeria A, clinical isolate described in this report.

Findings in the published literature vary in terms of use of artemisinin derivatives for in vitro drug susceptibility testing. Jambou et al. reported treatment failures with ACT in Cambodia, French Guiana, and Senegal (*8*). These authors used artemether for testing and showed IC_50_ values of ≈30 nmol/L in their “resistant” isolates from Senegal. Noedl et al. described treatment failures with ACT in Cambodia, for which IC_50_ values to dihydroartemisinin were ≈10 nmol/L (*12*). Dondorp et al. showed IC_50_ values of 4–6 nmol/L to dihydroartemisinin and 6–8 nmol/L to artesunate in a region of Cambodia and Thailand where ACT treatment failures have occurred (*4*). Systematic molecular surveillance and standardized drug-testing methods with clinical isolates are required to establish the molecular correlates of reduced susceptibility to antimalarial drugs. In this regard, efforts are ongoing under the auspices of the Worldwide Antimalarial Research Network (*13*).

## Conclusions

The patient’s infection responded to mefloquine when she was back in Canada, possibly because of the high oral dose of mefloquine. Current guidelines from the US Centers for Disease Control and Prevention recommend quinine sulfate plus doxycycline, tetracycline, or clindamycin; or atovaquone-proguanil (Malarone; GlaxoSmithKline, Mississauga, Ontario, Canada) as first- and second-line treatment for uncomplicated *P. falciparum* malaria. Reduced susceptibility to artesunate is more likely to occur when it is associated with inappropriate use of artemisinin derivatives than because of circulating artemisinin-resistant *P. falciparum* in sub-Saharan Africa.

In an effort to achieve consensus that artesunate oral monotherapies should not be marketed, the World Health Organization convened the international pharmaceutical sector in April 2006. At that time, 15 companies agreed to cease manufacturing artesunate monotherapies. However, oral artesunate montherapies may still be purchased over the counter in malaria-endemic countries, as this report shows. Thus, strains of *P. falciparum* malaria are currently at risk of developing reduced susceptibility to artesunate derivatives.
